# Diagnostic MALDI-TOF MS can differentiate between high and low toxic *Staphylococcus aureus* bacteraemia isolates as a predictor of patient outcome

**DOI:** 10.1099/mic.0.001223

**Published:** 2022-08-23

**Authors:** Tarcisio Brignoli, Mario Recker, Winnie W. Y. Lee, Tim Dong, Ranjeet Bhamber, Mahableshwar Albur, Philip Williams, Andrew W. Dowsey, Ruth C. Massey

**Affiliations:** ^1^​ School of Cellular and Molecular Medicine, University of Bristol, Bristol, BS8 1TD, UK; ^2^​ Centre for Ecology and Conservation, University of Exeter, Penryn, TR10 9FE, UK; ^3^​ Institute of Tropical Medicine, University of Tübingen, Tübingen, Germany; ^4^​ Department of Population Health Sciences and Bristol Veterinary School, University of Bristol, Bristol, BS8 2BN, UK; ^5^​ Severn Infection Sciences, North Bristol NHS Trust, UK; ^6^​ UK Health Security Agency, and University Hospitals Bristol & Weston NHS Trust; ^7^​ Schools of Microbiology and Medicine and APC Microbiome Ireland, UCC, Cork, Ireland

**Keywords:** Agr, bacteraemia, MALDI-TOF MS diagnosis, *Staphylococcus aureus*, toxicity

## Abstract

*
Staphylococcus aureus* bacteraemia (SAB) is a major cause of blood-stream infection (BSI) in both healthcare and community settings. While the underlying comorbidities of a patient significantly contributes to their susceptibility to and outcome following SAB, recent studies show the importance of the level of cytolytic toxin production by the infecting bacterium. In this study we demonstrate that this cytotoxicity can be determined directly from the diagnostic MALDI-TOF mass spectrum generated in a routine diagnostic laboratory. With further development this information could be used to guide the management and improve the outcomes for SAB patients.

## Background

*Staphylococcus aureus* is a major human pathogen that can cause a range of infections that vary in severity from superficial skin and soft tissue infections to fatal cases of pneumonia and bacteraemia [1, 2]. *S. aureus* bacteraemia (SAB) represent a significant burden of disease globally, with a 30 day mortality of approximately 20%, that has not substantially changed in over two decades [3]. This is despite general improvements to disease control (that have led to a decline in MRSA rates), improved laboratory diagnostics, and the availability of relatively newer classes of antimicrobial agents (e.g. linezolid, daptomycin and dalbavancin) over this time period. A greater understanding of the pathogenesis of SAB and more accurate pathogen-related diagnostics are urgently needed.

While SAB severity has been largely attributed to comorbidities of the host [2], specific *S. aureus* virulence factors have been highlighted in recent studies as making significant contributions to patient outcome following SAB [2, 4], in particular the ability of the bacteria to produce cytolytic toxins [5]. These toxins cause various clinical syndromes with a range of patient outcomes [1] and are a well-established means by which *S. aureus* transmits within and between hosts [6]. Although the *S. aureus* genome encodes a variable number of diverse cytolytic toxins, which are differentially distributed between lineages, one common feature across all *S. aureus* lineages is the regulation of the expression of these cytolytic toxin genes by the Accessory Gene Regulator (Agr) quorum sensing system [7]. Several studies focusing on the activity of the Agr system have shown a positive association between mutations that result in a dysfunctional Agr system and invasive diseases, such as SAB and pneumonia [5, 8, 9]. However, while such Agr-defective, non-toxigenic strains of *S. aureus* can cause SAB, the 30 day mortality rate is lower than those caused by strains with higher levels of cytolytic toxin production, when corrected for patient risk factors, such as age and co-morbidities [5]. It is therefore possible that the early differentiation between high and low toxicity cases of SAB could lead to improved interventions, including ICU review/admission and adjuvant protein synthesis inhibiting antibiotics with aim of inhibiting toxin production [10–16].

While most modern clinical diagnostic laboratories rely on multiple culture-based methods for the identification of pathogens and susceptibility testing, Matrix-Assisted Laser Desorption/Ionization-Time Of Flight Mass Spectroscopy (MALDI-TOF MS) technology is becoming the dominant method of identifying bacteria from primary cultures, including blood cultures [17, 18]. This proteomics-based technique has revolutionised diagnostics with its rapid turnaround times, where it produces a spectrum of the abundance of small (<10 kDa) proteins and lipoproteins produced by the cultured organism. The spectrum contains signature peaks used to identify the bacteria to a species level in a matter of minutes, depending on the platform used. There are however a huge number of peaks that are disregarded during the automated clinical MALDI-TOF MS analysis, and this disregarded data potentially contains a wealth of useful pathogen-related information. Given the importance of cytotoxicity to outcome following SAB, here we sought to determine whether this could be determined for individual bacterial isolates using standard diagnostic MALDI-TOF MS equipment, with a view to providing a more accurate characterisation of the bacterium causing SAB to aid with clinical management.

## Methods

### Bacterial strains and culturing

The laboratory strains used in this study are listed in Table 1. The clinical isolates and associated meta-data for each isolate (first described in reference 5) used in this study are listed in Table S1 (available in the online version of this article). All strains were cultured on Tryptic soy agar (TSA) plates at 37 °C for 24 h. Single colonies were smeared onto a clean MALDI plate, covered with 1 µl of HCCA matrix solution and once dried, samples were analysed using MALDI-TOF MS (Bruker MALDI Biotyper, Bruker Daltonics). As the instrument was in routine clinical use, instrument settings were fixed to the default clinical protocol which adapts scan time to terminate when the species was identified.

**Table 1.** Laboratory strains used in this study

Strain	Description	Reference
JE2	USA300; CA -MRSA, type IV SCC*mec*; lacking plasmids p01 and p03; wild-type strain of the NTML	[25]
JE2 *agr::tn*	*agr* transposon mutant in JE2	[25]
LAC	USA300; CA -MRSA, type IV SCC*mec*;	[22]
LAC Δ*hld*	LAC mutant in which the *hld* start codon has been altered in order to abolish δ-toxin production, but retains Agr activity.	[22]

### MALDI-TOF MS spectra extraction and analysis

To facilitate the computational analysis and comparison of MALDI-TOF MS spectra data, we first converted the absolute signal from each spectrum to relative values (relative to spectrum’s maximum peak) and subtracted the minimum over a moving window (0.005 × number of recorded peaks) such that the baseline across the entire spectrum was zero and its values ranged between 0 and 1. For peak extraction, we then selected the local maxima within non-overlapping 100 Da-windows across the spectrum above a relative peak height threshold of 0.02 (i.e. 2 % of the maximum peak) to form a peak-selected spectra for comparative analysis. Note, whereas the threshold value only affects the total number of selected peaks for downstream analysis, with no effect on the results presented here, the window size also affects the location assigned to the locally selected peaks and was chosen here to allow for optimal comparison between spectra of repeat samples or of spectra from different isolates. A visualised example of this approach is shown in Fig. S1. For each of the laboratory strains eight independent spectra were collected and analysed, and for the each of the clinical isolates four were collected and analysed. For the 3000 Da (Hld) peak the threshold for its presence was set at >0.1 relative peak height, based on the bimodal distribution in relative peak heights.

### Statistical analysis

We compared two logistic regression models fitted against clinical data of *S. aureus* bacteraemic patients with 30 day mortality as the (binary) response variable and (i) patient age +Charlson Comorbidity Index (CCI), or (ii) patient age +CCI+Pos3000 as the predictor variables, where Pos3000 refers to the presence or absence of a 3 000 Da MALDI-TOF MS peak. Model performances were assessed by means of the respective area under the ROC (receiver operating characteristic) curves (AUC), based on repeated (*N*=100) three-fold cross-validation. AUCs are reported as the mean across all repeats, with ranges provided as mean±standard deviation. All analyses were performed in R version 3.6.2 [19].

## Results

**Fig. 1.**
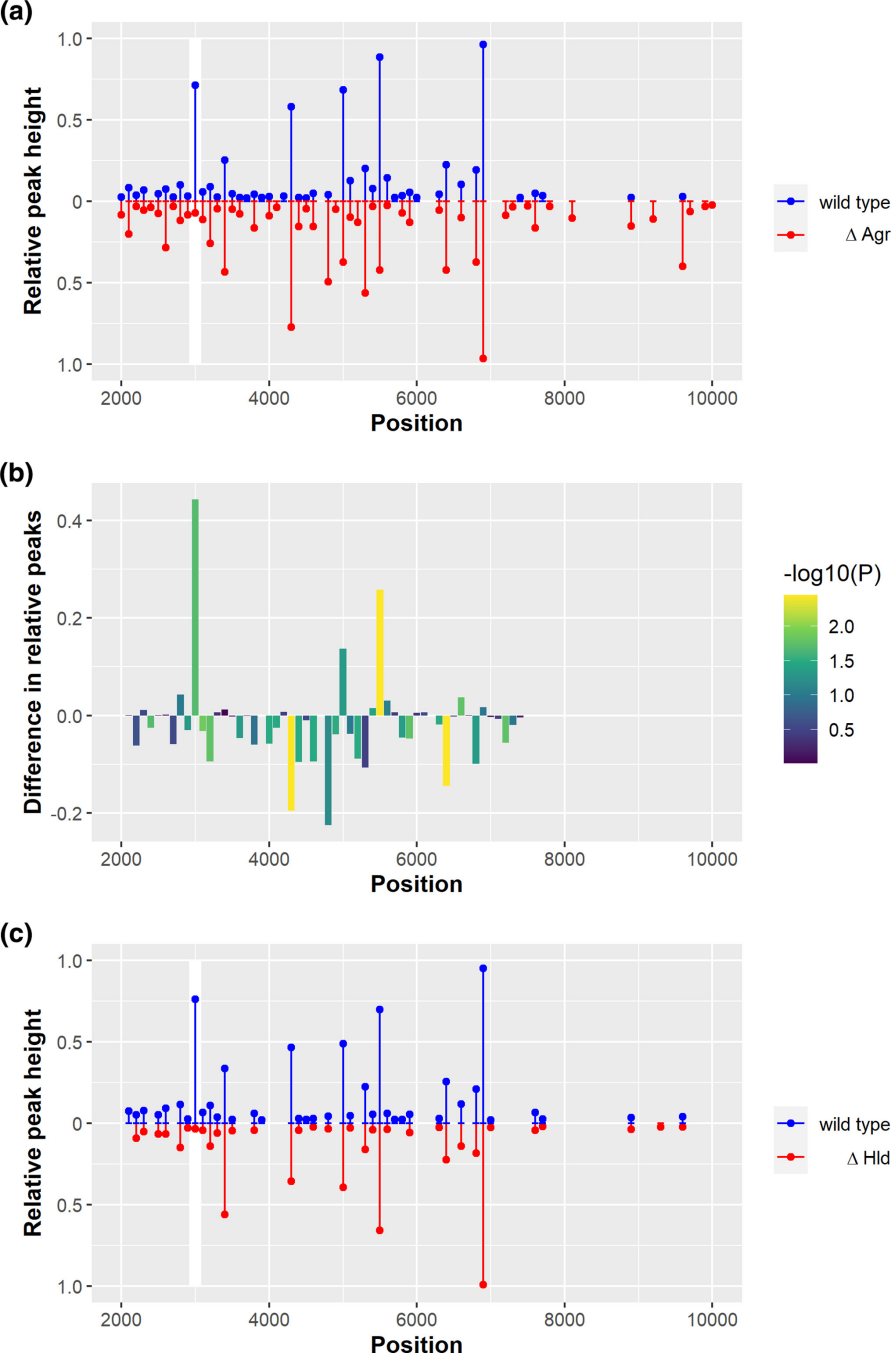
Diagnostic MALDI-TOF can differentiate between *S. aureus* isolates with and without a functional Agr system. (**a**) Comparison of relative spectra produced for an isogenic pair of a wild-type (blue) and an *agr* mutant strain (red), based on eight repeated runs, with the peaks at 3000 Da highlighted. (**b**) Average peak differences between the relative MALDI-TOF spectra of the wild-type and *agr* mutant, colour-coded by statistical significance of the difference in mean height (Welch two sample t-test, corrected for multiple testing using Benjamini-Hochberg with *N*(peaks)=53). A number of significant differences were detected with the biggest difference identified as the peak at 3000 Da. (**c**) Comparison of relative MALDI-TOF spectra of an isogenic pair of a wild-type and a Hld mutant strain identifying the 3000 Da peak (highlighted in white) as the Hld toxin.

The Agr two-component system is the major regulator of cytolytic toxin production by *S. aureus* [7]. It is frequently switched off through the mutation of key residues of the Agr proteins during the development of invasive disease [8, 9]. As the activity of the Agr system and the consequent effect on the cytolytic activity of the bacteria is a predictor of patient outcome following SAB [5], we sought here to examine whether diagnostic MALDI-TOF MS data could be used as a rapid means of quantifying this activity. For this we developed a computational approach to first extract dominant peaks from the full spectra then used these for comparative analyses. We used this approach to generate and analyse MALDI-TOF MS spectra for a wild-type *S. aureus* strain and an isogenic *agr* mutant using the standard operating procedure of the diagnostic laboratory [18]. Both strains produced spectra identified by the Bruker software as being *S. aureus *; however, we identified a number of peaks that were consistently different between the two (Fig. 1a, b).

One of the most significant differences between the spectra of the wild-type and Agr mutant was the peak at approximately 3 000 Da that was present in the wild-type strain and absent in the mutant (Fig. 1a, b). Delta toxin (Hld) is one of the major cytolytic toxins produced by *S. aureus* and is encoded within the *agr* locus, and previous work has detected this protein by whole cell MALDI-MS of clinical samples [7, 20]. A peculiarity of Hld is that its gene is encoded within the RNAIII molecule, which is the Agr regulatory molecule that switches on the expression of the other cytolytic toxins [7, 21]. So, in its RNA form it is the primary switch for cytolytic toxin production, and when translated it becomes itself a cytolytic toxin. As such, its expression is widely accepted as not only an indicator of the Agr activation status of the bacterium, but of the ‘gross’ cytolytic activity of the species. Hld is predicted to be approximately 3 000 Da, which suggests this 3 000 Da peak difference between the wild-type and *agr* mutant may be Hld. To verify this, we generated and analysed MALDI-TOF MS spectra for an isogenic wild-type and *hld* mutant pair of strains [22]. This *hld* mutant has had its start codon mutated, such that the Hld protein is not translated, but there is no effect on the transcription of the gene, or of the RNAIII molecule. As such, all other Agr regulated genes are still expressed as for a wild-type strain [22]. As illustrated in Fig. 1c, the only significant difference between these spectra is the peak at 3 000 Da. This work therefore clearly demonstrates that diagnostic MALDI-TOF MS spectra can be used to characterise Agr activity, and therefore potentially also the cytotoxicity of individual *S. aureus* isolates based on the Hld peak at 3 000 Da.

**Fig. 2.**
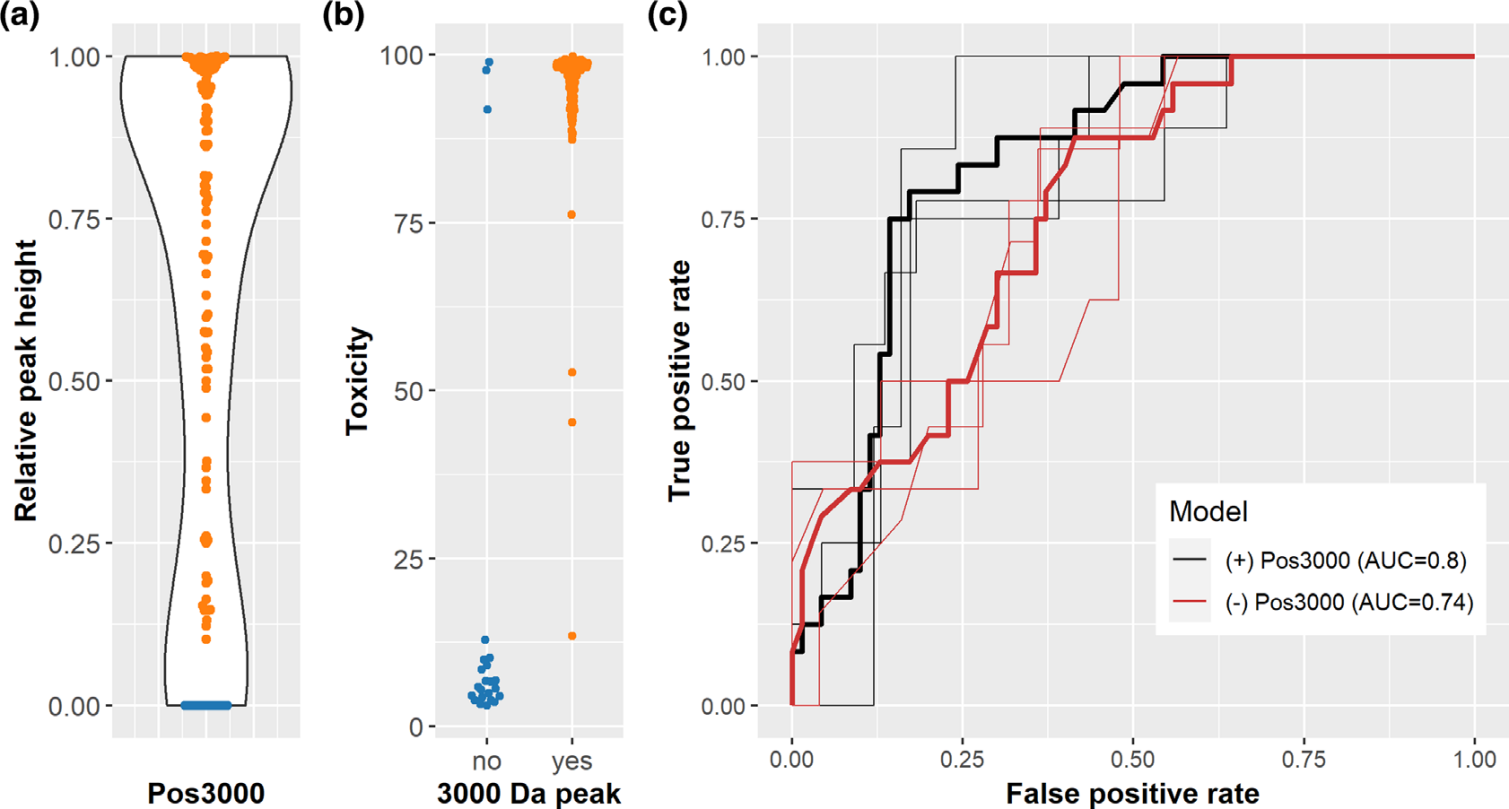
The 3000 Da peak is strongly associated with cytotoxicity and is predictive of 30 day mortality following SAB. (**a**) Relative peak heights at position 3 000 Da shows a bimodal distribution with some strains lacking this peak altogether (blue circles). (**b**) There is a significant association of the 3 000 Da peak and the cytotoxicity of 136 SAB isolates, as demonstrated by means of percentage of cell lysis (toxicity) stratified by presence or absence of the 3 000 Da peak. (**c**) Receiver operating characteristic (ROC) curves of fitted logistic regression models of patient age, patient comorbidity and the presence/absence of the 3 000 Da peak (black lines) compared with a model considering patient age and comorbidity alone (red lines), demonstrating improved discrimination of patient outcome (30 day mortality) when considering the toxicity-associated MALDI-TOF peak. Thick lines are based on the respective models fitted to all data; thin lines correspond to ROC curves based on a single run of a three-fold cross-validation, with reported AUCs based on 100 repeat runs.

To examine how applicable this approach is for scoring the toxicity of clinical isolates, we applied it to a collection of 136 *S *. *aureus* bacteraemia (SAB) isolates, for which we had clinical data pertaining to the patient, including their age, comorbidities, and 30 day mortality. The distribution in relative peak height at position 3000 in this set of strains was bimodal, with one set of strains lacking this peak altogether (Fig. 2a). For each isolate we had previously measured their cytotoxicity by incubating bacterial supernatant with cultured THP-1 progenitor monocyte cells, which is a cell type susceptible to the majority of cytolytic toxins *S. aureus* can produce [5]. As expected, the 3000 Da peak was strongly associated with the general cytotoxicity of the isolates (*P*<10^−10^, Welch two sample t-test). With only a few exceptions we found if there was a 3000 Da peak in the spectra for the isolate, they were highly cytolytic, and vice versa (Fig. 2a). A small number of isolates were outliers to this (indicated on Table S1), and we believe it is possible that given how genetically polymorphic the *agr* locus is, those that were cytolytic but with no 3 000 Da peak might be similar to the *hld* mutant described above in that they produce RNAIII, and as such have a functional Agr system, but do not produce any Hld protein.

In previous work we demonstrated that the cytolytic activity of individual SAB isolates was predictive of 30 day mortality, as was patient age and their Charleston Comorbidity Index (CCI) [5]. Other bacterial factors were also identified as predictive of patient outcome; however, as age, CCI and the 3 000 Da peak are immediately available to clinicians once a culture is confirmed as *S. aureus* by MALDI-TOF MS, we examined how well these three features predicted patient survival following SAB. For this we compared a logistic regression model for patient survival using patient age and CCI as explanatory variables with a model that also included the 3 000 Da peak. As illustrated by means of the corresponding ROC curves in Fig. 2b, the inclusion of the 3 000 Da peak leads to much improved model performance with respect to predicting patient survival rates (mean AUC=0.80[0.73, 0.87] versus mean AUC=0.74[0.66, 0.83] for the model without the 3 000 Da peak).

## Discussion

The ability to differentiate between fatal and survivable cases of SAB, at the point of diagnosis, has significant clinical implications. This can range from the preparation of the patient and their family for traumatic outcomes, to the application of targeted therapeutics that interfere with key aspect of the virulence of the causative agent [11, 12]. In the case of toxin production by *S. aureus, s*everal *in vitro* studies have demonstrated that antibiotics such as linezolid and clindamycin that act at the ribosome can suppress toxin production [11, 12]. Indeed, it has become standard clinical practice to add one of these antibiotics as adjuvant therapy in a range of clinical conditions including toxic shock syndrome, necrotizing fasciitis and PVL necrotizing pneumonia [13–16]. While the clinical benefit of this remains an area of some uncertainty, with limited clinical data in severe infections, clinical trials of toxin supressing adjuvant therapy in SAB are underway [23]. Given the proportion of SAB that are caused by Agr-defective, non-toxigenic *S. aureus* isolates, this could mask the benefit these therapies may have for highly toxic cases, as to date we have not been able to determine an isolate’s toxicity in a clinically relevant timescale. The findings presented here could help target the use of these adjunct toxin-suppressing therapies to toxigenic SAB cases with higher probabilities of a fatal outcome, and likewise prevent their ineffective use in non-toxigenic cases.

Current routine clinical diagnostic laboratories are solely focused on the identification of pathogens to species level, and their antimicrobial susceptibility with an acceptable turnaround time. This approach has not changed even with the exponential growth in the application of molecular and proteomics-based platforms in the recent years, where MALDI-TOF MS has now become the standard methodology in pathogen identification [17, 18]. Our study demonstrates how the detection of a specific virulence factor (e.g. the Hld 3 000 Da peak) produced by the causative pathogen in real time could potentially contribute to predicting the clinical outcome of patients and guide clinicians in the management of SAB.

There are a number of steps that would need to be taken before this could be implemented clinically. In this study we focussed on just one lineage (CC22) and did not include the second one (CC30) used in our original work [5]. Several factors contributed to this omission: that we had clinical data for only 77 CC30 infected patients; that the average toxicity of these isolates was low (described by us here [24]); that mortality was also low for this group despite the average age and comorbidities of this group being higher (relative to the CC22s). As such, the first step towards any clinical implementation of these findings would be to externally validate the results by expanding it to include a larger, more genetically and globally diverse collection of SAB, which is currently underway. In addition to demonstrating how applicable this approach may be, this broader study will also allow us to examine how diverse genetic background (e.g. lineage effects), or how different Agr types affect the MALDI-TOF MS spectra. Examining whether the Hld peak is as readily detectable if the bacteria are grown in broth will also be an important next step, as this becomes more common and timely in the diagnosis of SAB [17]. If successful we will then work with MALDI-TOF MS manufacturers to get the 3 000 Da peak included as part of their standard spectral analysis such that it informs clinicians on the toxicity of the infecting organisms at the same time as it identifies the organism as *S. aureus *. Although this is as yet at an early stage, this work has the potential to become a ‘game changer’ in not only SAB, but also other invasive infections caused by a range of pathogens, with considerable clinical implications in terms of prioritisation of care, targeted antimicrobial therapy and optimal resource utilisation in any healthcare system around the world.

## Supplementary Data

Supplementary material 1
