# Supplementary material for: Diagnostic MALDI-TOF MS can differentiate between high and low toxic Staphylococcus aureus bacteraemia isolates as a predictor of patient outcome
Source: Microbiology (Reading). 2022 Aug 23;168(8):001223. doi: 10.1099/mic.0.001223 (PMC10323763; doi:10.1099/mic.0.001223)
Supplement: Supplementary material 1 [file mic-168-1223-s001.pdf]

Supplementary Figure 1

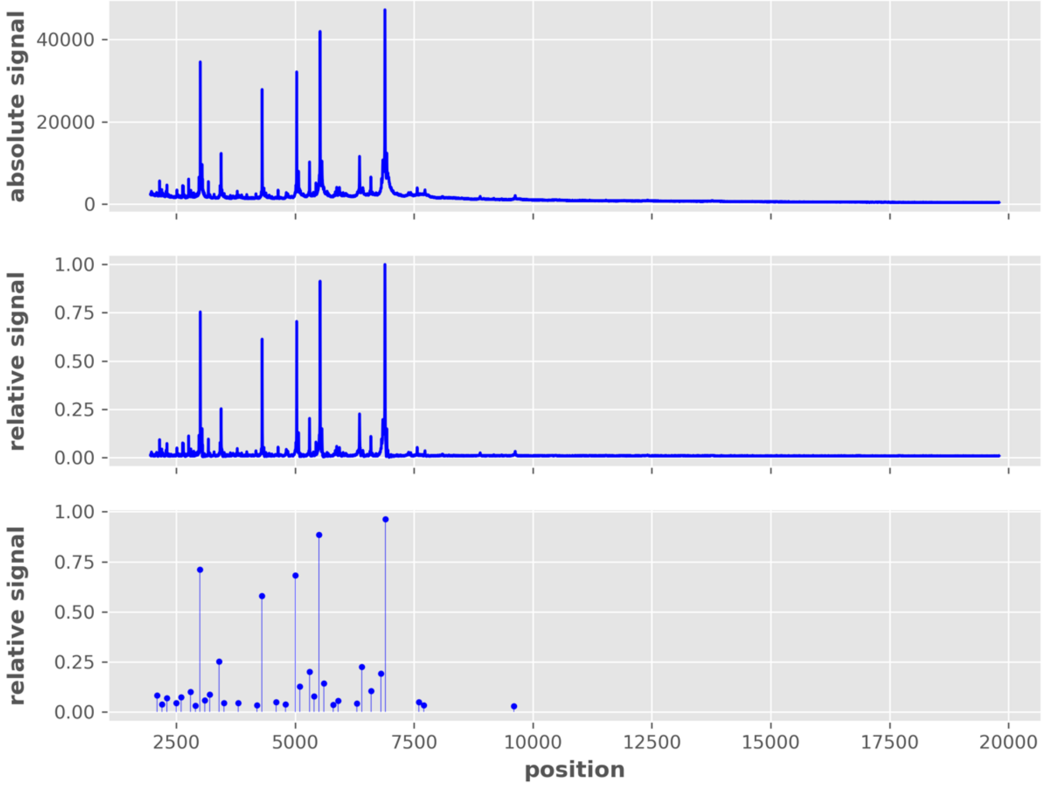

Supp. Fig. 1: MALDI-TOF spectra extraction and analysis A representative MALDI-TOF spectrum for a *S. aureus* isolate is provided (top) with the stages of a data analysis provided. The middle panel shows the affect the baseline subtraction has on the spectrum, and the bottom panel how the spectrum looks when the peaks are reduced a single maximum peak within each 100Da window.

Supp. Table 1: Clinical Isolates And Associated Metadata Used In This Study.

Strain ID	30 day outcome	Age	CCI	% toxicity
ASARM61	Alive	49	2	97.7
ASARM70	Alive	53	1	98.8
ASARM71	Alive	85	5	98
ASARM72	Alive	87	1	95.1
ASARM73	Death	51	6	98.6
ASARM74	Death	47	3	98.5

CCI: Charlston Co-morbidity Index
#N/A = data not available
Outliers

ASARM77	Alive	64	2	98.3
ASARM76	Death	76	14	98.6
ASARM75	#N/A	58	#N/A	98.3
ASARM80	Alive	74	2	97.5
ASARM79	Alive	71	2	97.8
ASARM59	Alive	34	1	5.7
ASARM83	Alive	23	2	97.7
ASARM84	#N/A	22	#N/A	5.4
ASARM86	Alive	74	4	3.9
ASARM87	Alive	73	7	4.6
ASARM89	Death	81	4	96.9
ASARM62	#N/A	66	#N/A	99.3
ASARM93	Alive	74	9	9.1
ASARM95	Alive	76	7	97.1
ASARM96	Alive	48	3	97.6
ASARM97	Alive	65	3	13.5
ASARM99	Death	87	4	76.2
ASARM100	Death	91	0	98.5
ASARM101	Death	71	3	98.6
ASARM102	Death	92	8	98.4
ASARM103	Death	90	8	98
ASARM105	Death	85	7	99.2
ASARM107	Alive	75	3	87.9
ASARM109	Alive	47	4	5.9
ASARM114	Death	66	11	98.5
ASARM64	Alive	74	2	96.4
ASARM116	Death	83	3	98.4
ASARM117	#N/A	75	#N/A	98.3
ASARM118	Alive	81	5	3.3
ASARM119	Death	83	#N/A	98.7
ASARM120	Alive	68	6	98.3
ASARM121	Alive	72	5	98.4
ASARM122	Alive	63	2	98.9
ASARM124	Alive	47	4	98.8
ASARM125	Death	86	5	98.4
ASARM126	Alive	89	2	3.9
ASARM65	Alive	66	2	4.4
ASARM127	#N/A	86	#N/A	98.3
ASARM128	Alive	70	4	3.1
ASARM132	Alive	29	1	97.7
ASARM133	Alive	86	0	90.9
ASARM134	Death	72	2	98.9

ASARM135	Alive	60	2	98.7
ASARM136	Death	83	0	96.9
ASARM137	Death	78	1	98.4
ASARM67	Alive	79	4	98.9
ASARM138	Death	60	6	99
ASARM139	Alive	54	6	99.2
ASARM140	#N/A	79	#N/A	98.5
ASARM141	#N/A	#N/A	#N/A	99.4
ASARM142	Alive	69	2	3.7
ASARM143	Alive	86	#N/A	91.9
ASARM144	Alive	61	2	98.6
ASARM145	#N/A	87	#N/A	96.8
ASARM68	Alive	88	8	98.2
ASARM148	#N/A	87	#N/A	98.1
ASARM154	Alive	75	6	12.9
ASARM153	Death	57	5	94.7
ASARM155	Alive	75	1	99.8
ASARM160	#N/A	56	#N/A	98.8
ASARM69	Alive	57	0	99.3
ASARM162	Alive	85	1	98.9
ASARM164	#N/A	73	#N/A	99
ASARM163	Alive	53	9	98.2
ASARM166	#N/A	45	#N/A	97.9
ASARM165	Alive	66	8	94.2
ASARM167	Alive	83	2	98.3
ASARM168	Death	65	6	98.5
ASARM169	Alive	96	2	98.7
ASARM179	Death	65	#N/A	98.3
ASARM181	Alive	64	#N/A	97.8
ASARM183	#N/A	62	#N/A	96.9
ASARM184	Alive	83	8	97.8
ASARM170	Alive	66	#N/A	91.7
ASARM191	Alive	75	9	97.9
ASARM193	Alive	44	8	98.4
ASARM196	Alive	0	#N/A	99.1
ASARM199	Alive	76	9	98.3
ASARM200	#N/A	74	#N/A	91.9
ASARM201	#N/A	51	#N/A	93.1
ASARM171	Alive	66	#N/A	95.5
ASARM203	Alive	4	#N/A	6.8
ASARM204	#N/A	85	#N/A	4.9
ASARM205	#N/A	66	#N/A	45.3

ASARM208	Alive	61	2	97.4
ASARM209	Alive	70	2	98.7
ASARM207	Alive	97	1	4.5
ASARM211	#N/A	88	#N/A	99.1
ASARM212	Alive	66	2	98.8
ASARM172	Death	88	4	97.4
ASARMLT1	Alive	66	#N/A	95.9
ASARMLT2	Alive	66	#N/A	94.5
ASARMLT3	Alive	66	#N/A	94.8
ASARM195	#N/A	91	#N/A	96.4
ASARM176	Alive	64	0	98.6
ASARM177	Death	89	0	98.6
ASARM217	#N/A	87	#N/A	91.9
ASARM220	Alive	55	#N/A	88.7
ASARM221	Alive	44	3	92.5
ASARM222	Alive	70	1	6.7
ASARM223	Death	55	#N/A	92.3
ASARM224	Alive	47	#N/A	90.9
ASARM110	Alive	59	2	98.3
ASARM108	Alive	46	0	92.2
ASASM42	Alive	37	0	98.9
ASASM56	Death	71	8	87.3
ASASM12	Alive	76	0	92.4
ASASM61	#N/A	#N/A	#N/A	91.4
ASASM64	#N/A	74	#N/A	93.7
ASASM71	Alive	81	4	92.6
ASASM73	Alive	65	1	96.5
ASASM90	Alive	70	2	52.7
ASASM96	#N/A	88	#N/A	6.7
ASASM97	Alive	37	#N/A	96
ASASM120	Alive	76	0	90.2
ASASM132	Alive	87	#N/A	95.8
ASASM138	Death	51	4	89.7
ASASM140	Alive	81	6	93.8
ASASM125	Alive	87	0	95.3
ASASM127	#N/A	#N/A	#N/A	95.2
ASASM181	Alive	76	2	90.2
ASASM186	Alive	64	9	93.4
ASASM190	Death	74	6	10.2
ASASM246	Alive	40	6	8.5
ASASM262	Alive	81	2	93.5
ASASM390	Alive	87	1	95.8

ASASM392	Alive	61	2	9.9
ASASM398	Alive	1	#N/A	88.3
ASASM430	Alive	51	1	95.2
ASASM168	#N/A	#N/A	#N/A	96.3
